# Polyclonal Broadly Neutralizing Antibody Activity Characterized by CD4 Binding Site and V3-Glycan Antibodies in a Subset of HIV-1 Virus Controllers

**DOI:** 10.3389/fimmu.2021.670561

**Published:** 2021-12-23

**Authors:** Tinashe E. Nyanhete, Robert J. Edwards, Celia C. LaBranche, Katayoun Mansouri, Amanda Eaton, S. Moses Dennison, Kevin O. Saunders, Derrick Goodman, Katarzyna Janowska, Rachel L. Spreng, Lu Zhang, Sarah V. Mudrak, Thomas J. Hope, Bhavna Hora, Todd Bradley, Ivelin S. Georgiev, David C. Montefiori, Priyamvada Acharya, Georgia D. Tomaras

**Affiliations:** ^1^ Center for Human Systems Immunology, Duke University School of Medicine, Durham, NC, United States; ^2^ Duke Human Vaccine Institute, Duke University School of Medicine, Durham, NC, United States; ^3^ Department of Immunology, Duke University School of Medicine, Durham, NC, United States; ^4^ Department of Medicine, Duke University School of Medicine, Durham, NC, United States; ^5^ Department of Surgery, Duke University School of Medicine, Durham, NC, United States; ^6^ Department of Cell and Molecular Biology, Feinberg School of Medicine, Northwestern University, Chicago, IL, United States; ^7^ Vanderbilt Vaccine Center, Vanderbilt University Medical Center, Nashville, TN, United States; ^8^ Department of Molecular Genetics and Microbiology, Duke University School of Medicine, Durham, NC, United States

**Keywords:** HIV-1 Virus Controllers, broadly neutralizing antibodies, antibody-dependent cellular phagocytosis (ADCP), CD4-binding site antibodies, negative-stain electron microscopy, neutralization fingerprinting assay

## Abstract

Broadly neutralizing antibodies (bNAbs), known to mediate immune control of HIV-1 infection, only develop in a small subset of HIV-1 infected individuals. Despite being traditionally associated with patients with high viral loads, bNAbs have also been observed in therapy naïve HIV-1+ patients naturally controlling virus replication [Virus Controllers (VCs)]. Thus, dissecting the bNAb response in VCs will provide key information about what constitutes an effective humoral response to natural HIV-1 infection. In this study, we identified a polyclonal bNAb response to natural HIV-1 infection targeting CD4 binding site (CD4bs), V3-glycan, gp120-gp41 interface and membrane-proximal external region (MPER) epitopes on the HIV-1 envelope (Env). The polyclonal antiviral antibody (Ab) response also included antibody-dependent cellular phagocytosis of clade AE, B and C viruses, consistent with both the Fv and Fc domain contributing to function. Sequence analysis of *envs* from one of the VCs revealed features consistent with potential immune pressure and virus escape from V3-glycan targeting bNAbs. Epitope mapping of the polyclonal bNAb response in VCs with bNAb activity highlighted the presence of gp120-gp41 interface and CD4bs antibody classes with similar binding profiles to known potent bNAbs. Thus, these findings reveal the induction of a broad and polyfunctional humoral response in VCs in response to natural HIV-1 infection.

## Introduction

The humoral responses in individuals naturally controlling HIV-1 [Virus Controllers (VCs)] include a polyclonal mix of antibodies that can mediate direct virus neutralization and antibody Fc effector functions [reviewed in (1, 2)]. Although neutralizing activity was identified in some virus controller or long term nonprogressor cohorts (3–5), other studies note a lack of or limited neutralization breadth in individuals naturally controlling HIV-1 replication (6–8).

Broadly neutralizing antibodies (bnAbs) are typically observed in non-controllers (viremic chronic progressors) with high viral loads and higher virus sequence diversity (9). The high viral loads and disease progression observed in non-controllers suggest that bnAbs are not associated with the control of autologous viruses and hence there is a perceived lack of association with protection against HIV-1 disease progression in VCs (7, 10, 11). However, there are a few isolated studies that have highlighted the presence of nAbs in patients with very low to undetectable viremia (12–17). Most importantly and unlike in chronic progressors, there is evidence from studies by Freund et al. (14) and Pilgrim et al. (17) showing that viruses isolated from VCs are sensitive to neutralization by autologous bnAbs. Epitope mapping of the VC bNAb response indicates predominant targeting of the V3-glycan supersite of vulnerability on the HIV-1 Envelope (Env) (14, 15). However, the epitope mapping of VC bNAb responses was done using approaches that limit the findings to already known bNAb targets defined using chronic viremic bNAb responses. The previous epitope mapping approaches have also largely overlooked poorly neutralizing or non-neutralizing antibody responses, which together with the neutralizing antibody response form part of the HIV-1 Env-directed immune response. Thus, there is a need for a comprehensive epitope mapping approach that assesses the polyclonal antibody response in patients mediating virus control. This approach will provide a more complete picture of the HIV-1 Env-directed immune response in the patient, and also has the potential to identify novel bNAb responses targeting previously unidentified epitopes in VCs.

Our work highlights a polyclonal bNAb response in VCs and provides the first evidence of the presence of an antibody response that is similar to a class of highly potent CD4bs bNAbs in a low viremia setting in the absence of autoimmunity. This work also adds to the growing literature supporting bNAb development in a low viremia setting that might be involved in mediating virus control.

## Materials and Methods

### HIV-1 Virus Controller Patient Cohort and Plasma samples

Thirty antiretroviral therapy (ART)-naïve HIV-1-infected VCs (maintaining plasma HIV-1 loads of <5,000 RNA copies/ml and CD4+ T cell counts of >400 cells/µl) and elite controllers (ECs) (maintaining plasma HIV-1 loads of <50 RNA copies/ml and CD4+ T cell counts of >400 cells/µl) (**Tables 1**, **2**) were enrolled through the Infectious Diseases Clinic at Duke University Medical Center upon these entry criteria. Patients were HLA typed using the next generation sequencing typing method (ProImmune Ltd, Oxford, UK).

**Table 1:** HIV-1 VC patient cohort.

Patient	Estimated Years Infected	Virus Load (copies/mL)	CD4 T Cell Count (cells/µL)	Protective HLA allele Status
VC N	~10	2170	821	None
VC O	~3	312	924	B57*03
VC Q	~7	2970	795	None
VC T	~3	1645^a^	430^a^	None
VC X	~2	601	627	B57*01
VC AA	~12	547	463	None
VC AB	~5	894	643	None
VC AC	~21	2060	997	B57*01
VC AD	~3	1040	449	B57*01
VC AH	~13	138	981	B57*03
VC AJ	~4	108	493	None
VC AK	~23	67	1825	B57*03
VC AL	~12	807^a^	748^a^	None
VC AM	~16	494	470	Not done^b^
VC AO	~12	299	972^a^	None
VC AP	~21	3496	776	B57*03
VC AQ	~24	300	1307	B27*05
VC AR	~20	222^a^	316^c^	B57*01
VC AT	~8	785	716	B57*03
VC AY	~10	64^a^	983^a^	None
VC BA	~11	4720^a^	757^a^	B57*03
VC BB	<1	487	466	B57*01

^a^indicates the viral load and/or CD4 count was not done for that draw date, and either an average of two data points from draw dates within 13 months or a single available data point within 13 months of that draw date based on available data. ^b^participant did not consent to genetic testing, ^c^from a sample draw date post enrollment.

Male and female HIV-1 infected patients not on ART and with a viral load above 50 and less than 5000 copies/mL of plasma and a CD4+ T cell count greater than 400 cells/µL of blood enrolled in the HIV-1 VC Cohort.

### TZM-bl Neutralization Assay

Neutralizing antibody activity in plasma samples was measured in 96-well culture plates using Tat-regulated luciferase (Luc) reporter gene expression to quantify reductions in virus infection in TZM-bl cells. Assays were performed with HIV-1 Env-pseudotyped viruses produced in 293T cells (**Supplemental Table 1**) as previously described (18). Plasma samples were heat-inactivated at 56°C for 30-minutes, then diluted over a range of 1:30 to 1:65610 in cell culture medium and pre-incubated with virus (~150,000 relative light unit equivalents) for 1-hour at 37°C before the addition of cells. Following a 48-hour incubation, cells were lysed, and Luc activity was determined using a microtiter plate luminometer and BriteLite Plus Reagent (Perkin Elmer). Neutralization titers are the sample dilution at which relative luminescence units (RLU) were reduced by 50% compared to RLU in virus control wells after subtraction of background RLU in cell control wells.

Mapping of the epitopes targeted by neutralizing antibodies in HIV-1 VC plasma samples with bNAb activity was performed with a panel of 4 viruses (TRO.11, COT6.15, JRFL and CH505TF) with mutations in the CD4bs, V2 glycan, V3 glycan, 2G12 and MPER epitopes targeted by bnAbs. A 3-fold decrease in the neutralization titer (ID_50_) for the mutant virus compared to the wild type virus indicated the involvement of that specific epitope in mediating neutralization of the wild type (parent) virus. A 3-fold change was used since it is outside of the normal variability of the assay (19).

### HIV-1-Specific Binding Antibody Multiplex Assay

HIV-1-specific antibodies in VC plasma were measured using the standardized HIV-1 binding antibody multiplex assay (BAMA) as previously described (20). HIV-1-specific antibody isotypes were detected with mouse anti-human IgG (Southern Biotech, Birmingham, AL) conjugated to phycoerythrin at 4 μg/ml. Antibody measurements were acquired on a Bio-Plex instrument (Bio-Rad, Hercules, CA), and the readout is mean fluorescent intensity (MFI). Antibody binding avidity was measured by BAMA with modifications as previously described (21).

BAMA epitope mapping was performed as previously described (20). IgG binding to the antigenically resurfaced gp120 glycoprotein RSC3 containing the CD4bs and to the RSC3Δ371I/P363N mutant (AIDS Research and Reference Reagent Program) was assessed. The RSC3Δ371I/P363N mutant has the isoleucine at position 371 of RSC3 removed to reduce b12 and VRC01 binding (22), which is further reduced by the P363N mutation (23). All assays were run under GCLP-compliant conditions, including tracking of positive controls by Levey-Jennings charts.

### THP-1 Antibody-Dependent Cellular Phagocytosis Assay

The ADCP assay was performed as previously described (24–26) using BG505gp140 T332N SOSIP.664 (27) and fluorescently labelled infectious virus particles (24). To calculate the phagocytosis score, a cutoff was first assigned based on the 95^th^ percentile of the no-antibody control. For each sample, the proportion of cells above this cutoff was multiplied by their mean fluorescence intensity (MFI), and then normalized to the corresponding result for the no-antibody control to give the final phagocytosis score. A background level of phagocytosis was determined based on the mean + 3 standard deviations of the non-HIV-1 specific antibody (CH65) (28).

### Infectious Virion Capture Assay

The infectious virion capture assay was performed as previously described (29). Briefly, 100 μl of a 20 μg/ml monoclonal IgG stock was mixed with 100 μl of diluted HIV-1_BaL_, HIV-1_CM235_ or HIV-1_1086C_ fluorescent virus in a 96-well 1.1 ml plate, and the plate was centrifuged at 450g for 90 minutes at room temperature to generate IgG-HIV-1 virion immune complexes (IC). A total of 150 μl of the immune complex was transferred onto a protein G plate attached to a new 96-well 1.1 ml receiving plate, and the plate was shaken at 800 rpm for 1 hour at room temperature. After the incubation, the plate was centrifuged for 3 minutes at 700g at room temperature, followed by 2 washes with 200μl 1X TBS to collect all the uncaptured immune complexes (flow-through) in the receiving plate. Qualitative measurement of the number of virions in the flow-through (un-captured virions) was done using the TZM-bl infection assay. A total of 25μl of virus from the receiving plate (flow-through plate) was used to infect 100μl of 16x10^4^ cells/ml freshly trypsinized TZM-bl cells. Controls included RPMI only (no-antibody) plus cells, and virus (no-antibody) plus cells. Infection was measured by firefly luciferase assay at 48-hours post-infection (at 37°C) as previously described (30). The percentage of infectious captured virus from the TZM-bl infection was calculated as follows: % of un-captured infectious virus (flow-through) = [(mean relative light units of sample)/(mean relative light units of no-antibody (RPMI) only)] * 100. The % captured infectious virus = 100 - % of un-captured infectious virus. The positivity cut-off was calculated as 3 times the no-antibody (RPMI) % captured infectious virus.

### Bio-Layer Interferometry Assay

Bio-layer interferometry (BLI) measurements were done with the ForteBio Octet RED 384 instrument and ForteBio streptavidin biosensors using the previously described protocol (31). Kinetics assays were performed at 25°C using the Standard Kinetics Acquisition rate (5.0 Hz, averaging by 20) at a sample plate shake speed of 1000 rpm. Biotinylated RSC3, BG505gp140 T332N SOSIP and BSA proteins were loaded onto streptavidin (SA) sensors, which were then dipped in kinetics buffer (ForteBio) to establish a baseline time course and then dipped into wells containing HIV-1 VC IgG or Fabs (diluted in kinetics buffer to 200 μg/ml) to monitor antibody association. The dissociation step was monitored by dipping antibody-bound sensors back into the wells used to collect the baseline time course. To subtract binding due to non-specific interactions of antibodies with the sensors, biotinylated RSC3Δ371/P363N – loaded SA sensors and biotinylated BSA-loaded SA sensors were used as controls for the RSC3 and the BG505gp140 T332N SOSIP, respectively.

### Neutralization Fingerprinting Analysis

Neutralization fingerprinting of the polyclonal antibody response in HIV-1 VCs N, AA, AL, AP, AQ and BA was performed using an optimized panel of 30 diverse strains (**Supplemental Table 2**) as previously described (32). Briefly, the neutralization fingerprint of a plasma sample is represented as a combination of the neutralization fingerprints of a reference set of bnAbs, grouped in ten epitope-specific clusters. The prevalence of each of the ten antibody groups can be estimated for the given plasma sample, with prevalence scores ranging between 0 (low) and 1 (high), with the positivity cut-off set at 0.25. Additionally, two measures (Residual score and Median of scores) are computed as a way to estimate prediction confidence for the prevalence scores (32).

### Negative-Stain Electron Microscopy

BG505gp140 T332N SOSIP trimers were incubated with purified HIV-1 VC Fab at molar ratios of 1:84 (VCAL), 1:60 (VCAP) and 1:49 (VCAQ) overnight at room temperature. After overnight incubation, the complexes were cross-linked with 7.5mM glutaraldehyde for 5 minutes at room temperature, and the excess glutaraldehyde was quenched with 75mM Tris buffer (pH 7.4) for 5 minutes at room temperature. The cross-linked Fab-Env complexes were then purified by loading them on a Superdex 6 increase 10/300 column using a 500 μl loop and run at 0.5 ml/min using an Akta Pure System (GE Healthcare). For negative stain, the complex diluted to 100 µg/ml with 5 g/dl Glycerol in HEPES buffered saline (HBS), containing 20 mM HEPES pH 7.4 and 150 mM NaCl. 5 µl of sample was applied to a glow-discharged carbon-coated EM grid for 10-12 second, then blotted, and stained with 2 g/dL uranyl formate for 1 min, blotted and air-dried. Grids were examined on a Philips EM420 electron microscope operating at 120 kV and nominal magnification of 82,000x, and images were collected on a 4MpixCCD camera at 4.02Å/pixel. Three-dimensional analysis of images of SEC-purified Fab-SOSIP complexes was performed using standard methods within the Relion 3.0 pipeline (33). Atomic models of SOSIPs with a single Fab bound were generated from published structures and fit as rigid bodies into the EM reconstructions using the Fitmap function of UCSF Chimera. The Fitmap function reports the number of atoms falling outside the map, and this was used as a relative measure of goodness of fit with a smaller number indicating better fit for a specific model. A similar approach was used to examine the gp41/gp120 interface Fab-SOSIP complex observed in VC AL.

### Autologous HIV-1 Sequence Isolation

Single-genome amplification (SGA) of cDNA for the 3’half of the genome from plasma viral RNA from virus controllers was performed as previously described (34). Sequence alignments and phylogenetic trees were constructed using CLUSTAL W (35) and adjusted manually when necessary using SEAVIEW (36). Phylogenetic trees were constructed using the neighbor-joining (NJ) method with the Kimura two-parameter model (37, 38). Highlighter plots were generated using the Highlighter tool at the Los Alamos HIV sequence database (https://www.hiv.lanl.gov/content/sequence/HIGHLIGHT/highlighter_top.html).

### Statistical Methods

Statistical analyses were performed using R statistical software (R Foundation for Statistical Computing, Vienna, Austria). All p-values are two-sided and considered significant at the 0.05 level. Neutralization breadth was defined as the number of isolates neutralized. ADCP breadth was defined as the average ADCP score across 3 viruses: BaL, CM235, and 1086C.

A magnitude-breadth (M-B) curve describes the magnitude (neutralization titer) and breadth (proportion of isolates neutralized) of an individual sample against a panel of HIV-1 isolates, while the area under the curve (AUC) provides a summary value of the M-B profile (39).

Correlations between neutralization breadth, ADCP breadth and estimated duration of infection were assessed using Spearman’s rank correlation. The correlation coefficients and associated p-values were obtained using the cor.test function in R.

Hierarchical clustering analysis was performed in R Studio using the pvclust package as previously described (https://github.com/shimo-lab/pvclust). Rectangles were drawn around branches with approximately unbiased p<0.05.

## Results

### A Subset of VCs Develops Broadly Neutralizing Antibodies

**Figure 1:**
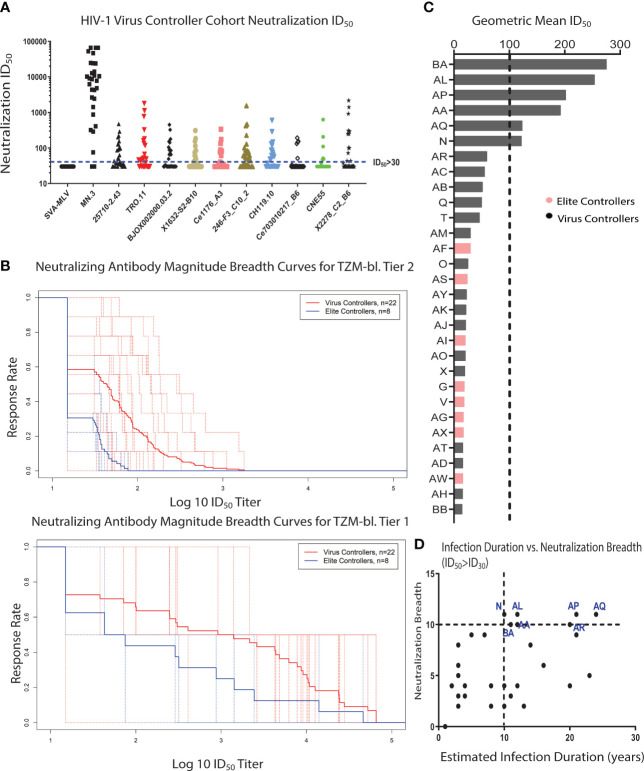
A subset of clade-B HIV-1 VCs develops bNAbs. **(A)** A global virus panel of 9 tier 2 viruses, 1 tier 1B virus, and 1 tier 1A virus (MN.3) was used to measure the magnitude and breadth of neutralizing antibodies (nAbs) in 30 HIV-1 Virus and Elite Controller plasma samples using a standardized TZM-bl neutralization assay. Murine Leukemia Virus (MLV) was used as the negative control. The cut-off for a positive neutralization signal against the tested virus was ID_50_ > 30 based on the criterion of a signal greater than or equal to three times that against the negative control MLV-pseudotyped virus. Each plasma sample was tested in triplicate against each virus in the panel. **(B)** The nAb magnitude and breadth against tier 1 and tier 2 viruses for VCs and ECs were compared using the Magnitude-Breadth (M-B) Curve (Tier 1 viruses Mean AUC: VCs = 2.869 and ECs = 2.216, Tier 2 viruses Mean AUC: VCs = 1.653 and ECs = 1.311). The solid line represents the means of all VE or EC participants, and each dotted lines represents an individual participant. **(C)** HIV-1 VC plasma samples with bNAb activity were identified using the bNAb criteria of having a geometric mean ID_50_ >100 and also neutralizing 90% of the viruses in the neutralization virus panel with an ID_50_ of at least 100. All the patients above the black dotted line fit the bNAb criteria. **(D)** Spearman correlation analysis of the neutralization breadth (the number of viruses neutralized with an ID_50_ of at least 30) and the estimated duration of infection based on the estimated date of diagnosis (r = 0.465, p = 0.0084). The HIV-1 VC patients in blue are the patients with bNAb activity, and they have all been infected for at least 10 years.

Plasma from VCs and ECs were tested for the presence of HIV-1 neutralizing antibodies (nAbs) with the TZM-bl nAb assay against a standardized panel of one tier 1A reference strain, one tier 1B strain, and nine tier 2 Env-pseudotyped HIV-1 viruses of multiple sub-types (**Supplemental Table 1**) (40) (**Figure 1A**).

Twenty-nine of the thirty patients neutralized the tier 1A strain (MN.3), while neutralization of the more difficult-to-neutralize tier 1B and 2 strains was limited to only a subset of the VC and EC patient cohort (**Figure 1A**). Neutralization breadth was defined as the fraction of Env-pseudotyped HIV-1 viruses (tier 1 and tier 2 strains) neutralized with an inhibitory dose 50 (ID_50_) titer greater than 30 (cut-off for positivity), while neutralization magnitude was defined as the natural logarithm of inhibitory dose 50 (ID_50_ titer). Quantification of the neutralization magnitude and breadth in the VC cohort using the magnitude-breadth curve (M-B curve) (39, 41) showed that patients with a relatively higher HIV-1 plasma viral load in this cohort had a higher neutralization magnitude and breadth response compared to patients with undetectable HIV-1 plasma viral load (ECs), which was more pronounced for tier 1 viruses (**Figure 1B**).

Polyclonal antibodies from participants in the VC and EC patient cohort also demonstrated antibody-dependent cellular phagocytic (ADCP) activity against a panel of clade A/E, B and C viruses and virion capture of infectious virus particles. Both results demonstrate the broad recognition of virus particles by the antigen binding site of the antibodies (**Supplementary Figures 1A, B**). The ADCP measurement also tests the capacity of the antibody Fc to interact with cellular Fc receptors, in addition to recognition of virus particles. ADCP breadth significantly correlated with neutralization breadth (rho = 0.46, p-value = 0.011) (**Supplementary Figure 2A**). However, the rho value indicates that the two immune parameters are related but not directly overlapping. The ADCP breadth/neutralization breadth correlational analysis revealed the presence of 2 clusters: high responders (high ADCP and high neutralization breadth) and low responders (high ADCP and low neutralization breadth) (**Supplementary Figure 2A, B**).

A subset of the patients with nAb activity against both tier 1 and 2 viruses developed broadly neutralizing activity (6/30) (**Figure 1C**), which is defined as being able to neutralize 90% of the strains in the global panel with a geometric mean ID_50_ greater than or equal to 100 (42, 43). All 6 patients with bNAb activity were from the VC cohort. The frequency of patients with bNAbs in this cohort (about 20%) was comparable to previously reported frequencies observed in another VC patient cohort (Boston area) (44) and a cohort of chronically infected patients with very high viral loads (8, 45). All the VC patients with bNAb activity were infected for at least 10 years (**Figure 1D**). Several participants in this study had known protective HLA alleles (**Table 2**) suggesting that robust HIV-1 CD8 T cell activity might also have been present within virus controllers (46–49). Taken together, the nAb analysis revealed the development of bNAbs in a subset of VCs with both Fc (ADCP)- and Fab (neutralization)-mediated antibody responses.

**Table 2:** HIV-1 EC Patient cohort.

Patient	Years Infected	Virus Load	CD4 T Cell Count	Protective HLA Allele Status
VC G	~8	47	1887	B57*03
VC V	~3	48	1847	B27*05
VC AF	~14	47	729	A32*01
VC AG	~4	47	1420	None
VC AI	~20	<20	1159	B57*03
VC AS	~3	<20	1093	None
VC AW	~10	<20	337^a^	Not done
VC AX	~11	<20	910	B57*01

a from a sample draw date post study enrollment. Male and female HIV-1 infected patients with a viral load less than 50 copies/mL of plasma and a CD4+ T cell count greater than 400 cells/µL of blood enrolled in the HIV-1 EC cohort.

### VCs With bNAb Activity Show Capacity to Internalize Native-Like Trimeric HIV-1 Env Glycoproteins

**Figure 2:**
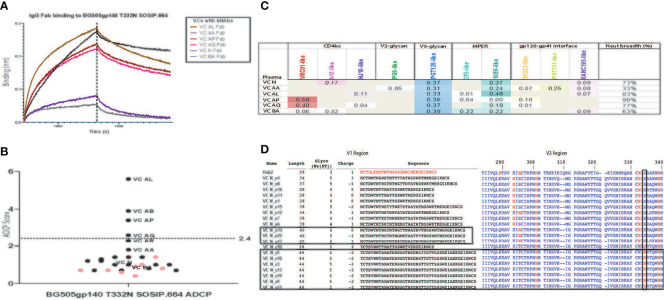
Polyclonal Fabs from HIV-1 VCs with bNAb activity target multiple vulnerable HIV-1 Env epitopes. **(A)** Fab fragments (200 μg/ml) from VCs with bNAb activity bind to BG505/T332N SOSIP.664 immobilized to streptavidin biosensors in a BLI assay. Fabs from VCs without bnAb activity, Fabs from an HIV-1 negative donor (seronegative Fab) and a kinetics buffer solution (negative control) did not bind (lack of visible sensorgram trace). BSA immobilized to the streptavidin biosensor was used for reference (background) subtractions. This assay was done in duplicate. **(B)** The ability of purified IgG from HIV-1 VCs (black dots) and ECs (pink dots) to form immune complexes with and mediate ADCP of BG505/T332N SOSIP.664 conjugated to 1um fluorescent beads was measured in a THP-1 ADCP assay. The bNAbs PGT125, PGT145, PGT151 and VRC01 were included as positive controls, while the non-neutralizing mAb F105 and CH65 were included as negative controls. Positive ADCP signal (above the blue dotted line) was calculated as 3X the ADCP score for the negative control CH65 mAb which was a score of 0.8. The assay was run in duplicate (%CV < 35% between replicates). **(C)** A Neutralization Fingerprinting (NFP) algorithm was used to predict the plasma neutralization pattern of the HIV-1 VCs with bNAb activity against the panel of diverse HIV-1 isolates shown in Table. For each HIV-1 VC plasma sample, the predicted prevalence of each of the reference bNAb clusters is shown as a score ranging from 0 – 1, where scores greater than or equal to 0.25 signals the presence of a neutralization pattern by the bNAb cluster and scores closer to 1 represent stronger neutralization signal by the bNAb cluster. Breadth represents the percentage of viruses neutralized with an ID_50_ > 40. The neutralization assay was performed in triplicate. **(D)** Comparison of HIV-1 *env* V1 sequences isolated from the plasma of HIV-1 VC N to the HXB2 (K03455) reference *env* V1 region shows an elongated V1 domain in a subset of the sequences from HIV-1 VC N (10/20 sequences). Alignment of the isolated HIV-1 VC N *env* V1 region sequences against the HXB2 reference was done using the LANL Sequence database sequence HIValign, and the V1 region sequence analysis was done using the Variable Region Characteristics program on the LANL Sequence database. Comparison of HIV-1 *env* V3 sequences isolated from the plasma of HIV-1 VC N to the HXB2 (K03455) reference *env* V3 region shows 8/20 sequences possessing the less frequent N334 glycan instead of the frequent N332 glycan. Analysis of the V3 region of the isolated 20 HIV-1 VC N *env* V3 region sequences was done using the LANL Sequence database tool Variable Region Characteristics.

Given that antibody binding to the trimeric HIV-1 Env (SOSIP) is a predictor of functional IgG nAb responses (50, 51), we measured the polyclonal Fab interactions with recombinant trimeric HIV-1 Env (BG505gp140 T332N SOSIP.664). We tested the sensitivity of the HIV-1 pseudotyped virus corresponding to 3 commonly used SOSIP constructs to neutralization by the VC plasmas with bNAb activity (**Table 3**) and proceeded with the well characterized BG505gp140 T332N SOSIP.664 for structure based analysis after confirming antigenicity by BLI. Prior to measuring SOSIP interactions with HIV-1 specific IgG from VCs with and without bNAbs, we first confirmed that immobilization of the biotinylated BG505gp140 T332N SOSIP.664 on streptavidin BLI biosensors does not result in conformational changes of the SOSIP and exposure of non-neutralizing epitopes as indicated by lack of binding of non-neutralizing mAbs (19b and F105) to the immobilized BG505gp140 T332N SOSIP.664 (**Supplementary Figure 3**). Interrogation of VCs with bNAb activity (VC AL, VC AA, VC AP, VC AQ, VC N and VC BA) and those without bNAb activity (VC AI, VC AW, VC AX, and VC BB), revealed that only VCs with bNAb activity exhibit Fab binding to the BG505gp140 T332N SOSIP.664, while no binding to the SOSIP is observed in VCs with no bNAb activity (**Figure 2A**).

**Table 3:** Neutralization sensitivity of the HIV-1 pseudotyped viruses with sequences corresponding to leading SOSIP constructs.

Patient	ID_50_ in TZM-bl Cells			
	MLV	CH505TF	BG505 T332N	JR-FL
VC N	<20	**44**	**158**	**268**
VC AA	<20	**68**	**64**	**255**
VC AL	<20	**51**	**175**	**2551**
VC AP	<20	**738**	**418**	**3191**
VC AQ	<20	**99**	**202**	**337**
VC BA	<20	<20	**118**	**85**

The neutralization sensitivities of the CH505TF, BG505/T332N and JR-FL HIV-1 pseudotyped viruses to HIV-1 VC plasma samples with bnAb activity were measured in a TZM-bl neutralization assay, with MLV being used as the negative control. Values in black bold type are considered positive for neutralizing antibody activity in the sample based on the criterion of a signal greater than or equal to three times that against the negative control MLV-pseudotyped virus. The CH01-31 mAb was used as the positive control (IC_50_ for CH505TF = 0.08, BG505 T332 = 0.06 and JR-FL = 0.04). Values are plasma dilution at which relative luminescence units (RLUs) were reduced 50% compared to virus control wells (no test sample). Each HIV-1 VC plasma sample was tested in triplicate.

To investigate the presence of any downstream effector function after binding to the native-like trimeric HIV-1 Env glycoproteins (SOSIPs), we tested the ability of IgG from the samples described above to mediate phagocytosis of the clade-mismatched native-like trimeric HIV-1 Env glycoprotein BG505gp140 T332N SOSIP.664 trimers. Three of the 4 VCs (VC AP, VC AL and VCAQ) that mediated internalization of the trimeric HIV-1 Env glycoprotein were VCs that displayed binding to the native-like trimeric HIV-1 Env glycoprotein and also had bNAb activity (**Figure 2B**). In contrast, none of the VCs without bnAb activity mediated ADCP. This further demonstrated that antibodies from a subset of patients with bNAb activity can both bind to the HIV-1 Env and mediate phagocytosis of the bound target. Taken together, the IgG and Fab binding data show that binding to native-like trimeric HIV-1 Env glycoprotein BG505gp140 T332N SOSIP.664 can distinguish VCs with and without bNAb activity.

### Serum Antibodies From VCs With bNAb Activity Bind to Conserved Vulnerable Sites on the HIV-1 Env

Given that the VC patients with bNAb activity (**Figure 1C**) displayed binding to the recombinant trimeric HIV-1 Env, BG505gp140 T332N SOSIP.664 (**Figure 2A**), we hypothesized that this binding indicated the presence of bNAbs targeting the HIV-1 Env. To determine whether the polyclonal antibody response from VCs with bNAb activity targeted previously characterized vulnerable bNAb epitopes, we performed epitope mapping of the polyclonal plasma and purified IgG from these patients.

Mapping of the polyclonal HIV-1 antibody response was first performed with the neutralization fingerprinting (NFP) assay (32) that predicts epitope specificities of polyclonal antibody responses to HIV-1 infection. This computational approach predicted the prevalence of PGT128-like (V3-glycan), 10E8-like (MPER) and VRC01-like (CD4bs) bNAb responses. The polyclonal antibody response was characterized by the targeting of multiple bNAb specificities in 5 of the 6 patients – VC N (V3-glycan and MPER), VC AA (V3-glycan, MPER and gp120-gp41 interface), VC AL (V3-glycan and MPER), VC AP (CD4bs and V3-glycan), VC AQ (CD4bs and V3-glycan), and VC BA (V3-glycan only) (**Figure 2C**). The presence of VRC01-like bNAb activity in some of the VC patients (VC AP and VC AQ) was also confirmed by measuring the IgG differential binding to RSC3 and RSC3δ371l/P363N using BAMA (**Table 4**).

**Table 4:** HIV-1 VC high responders possess VRC01-like CD4bs antibodies.

Sample	Cluster	WT RSC3	RSC3 Mutant	WT/Mutant Ratio
**VC N**	HR	630	100	**6.3**
VC AA	HR	216	377	0.6
**VC AL**	HR	2597	581	**4.5**
**VC AP**	HR	1236	142	**8.7**
**VC AQ**	HR	10741	100	**107.4**
**VC BA**	HR	625	100	**6.3**
VC AI	LR	100	100	1
VC AW	LR	304	316	1
VC AX	LR	100	100	1
VC BB	LR	100	100	1
**VRC01**	CD4bs bnAb	27247	114	**239**

Differential binding of HIV-1 VC IgG from a subset of high responders (HR) and low responders (LR) to wild type (WT) RSC3 and RSC3δ371l/P363N, which has mutations that diminish binding to VRC01 and b12. A differential binding ratio >2.5 (in black bold) represents the presence of VRC01 or b12-like CD4bs antibodies.

An unusually elongated V1 region (52–55) and an N332 to N334 glycan switch (54) have been shown to mediate escape from V3-glycan targeting bnAbs by potentially shielding and blocking access to the vulnerable V3-glycan epitope. Given the predominant targeting of the V3-glycan by the HIV-1 VC plasma, we sought to investigate whether this V3-glycan targeting resulted in autologous virus escape from the V3-glycan directed bnAbs by investigating the presence of the above highlighted modes of immune escape, N332 to N334 glycan shift and elongation of the V1 region.

Autologous HIV-1 *env* sequences were successfully isolated from the plasma (10 years from the time of infection) of one of the six HIV-1 VCs (VC N) with bNAb activity targeting the V3-glycan. V1 region sequence analysis revealed that 50% (10/20) of the isolated *env* sequences from VC N had unusually long V1 regions ranging from 39 – 44 amino acids in length (11 – 15 amino acids longer than the HXB2 reference, **Figure 2D**). The average V1 region length from the LANL HIV Sequence database is 27 amino acids, with 95% of the V1 region sequence length falling within the range of 15 – 39 amino acids (52). In addition, 40% (8/20) of the VC N *env* sequences had the N332 to N334 glycan shift (**Figure 2D**). Seven of the eight VC N *env* sequences with the N332 to N334 glycan shift were among those with the longest V1 region (44 amino acids) among all the isolated 20 *env* sequences (**Figure 2D**).

**Figure 3:**
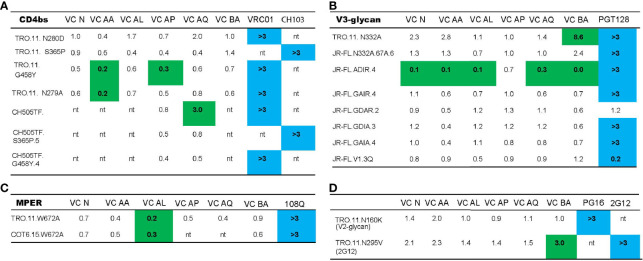
Neutralization breadth in VCs with bNAb activity is mediated by the polyclonal targeting of multiple bNAb epitopes. The ability of HIV-1 VC plasma to neutralize TRO.11, CH505TF, COT6.15 and JR-FL wild type viruses, together with their mutant counterparts - CD4bs mutants **(A)**, MPER mutants **(B)**, V3-glycan mutants **(C)**, V2-glycan mutants **(D)** and 2G12 epitope mutants **(D)** - was assessed in a TZM-bl neutralization assay. Control mAbs used were: VRC01 (CD4bs epitope), CH103 (CD4bs epitope), 10E8 (MPER epitope), PGT128 (V3-glycan epitope), PG16 (V2-glycan epitope), and 2G12 (2G12 epitope). Neutralization scores for VC plasma were measured as ID_50_, while the scores for the mAbs were measured as IC_50_. For VC plasma samples, the WT/Mutant ID_50_ values are reported, with changes in neutralization titer (ID_50_) more than three-fold denoted in green. Three-fold changes higher in the mutant (less than 0.33) compared to wild-type indicate a gain in neutralization sensitivity, and a three-fold change lower in the mutant (greater than 3) indicates a loss in neutralization sensitivity. For the mAbs, Mutant IC_50_/WT values are reported, with a three-fold change in IC_50_ (denoted in blue) indicating a loss (greater than 3) or gain (less than 0.33) in neutralization sensitivity as a result of the corresponding mutation. “nt indicates that the VC plasma or mAb was not tested with the virus based on previous neutralization fingerprinting analysis.

To investigate the functional relevance of targeting of the vulnerable epitopes of interest highlighted by the neutralization fingerprinting assay (**Figure 2C**), we mapped the nAb specificities in plasma samples that neutralized the parent virus with a titer ID_50_ >30. Based on this criteria, the parent viruses TRO.11 (tier 2), COT6 (tier 2), JR-FL (tier 2) and CH505TF (tier 2) were employed for the epitope mapping TZM-bl neutralization assay, as these viruses had corresponding variants with mapping mutations in the CD4bs (**Figure 3A**), MPER (**Figure 3B**), V3-glycan (**Figure 3C**), and glycan epitopes (**Figure 3D**) known to be targeted by bnAbs (56, 57). A three-fold decrease in the neutralization titer (ID_50_) (outside the normal variability of the assay) against the mutant compared to the wild-type parent virus indicates dependency on that mutation for neutralization sensitivity (19). For all the wild-type parent/mutant variants pairings, only the mutation in the V3-glycan epitope (N332A) in the TRO.11 virus resulted in at least a 3-fold decrease in neutralization titer for VC BA (**Figure 3C**). None of the other tested epitope mutations in the parent viruses resulted in loss of neutralization sensitivity to VC N, VC AA, VC AL, VC AP or VC AQ plasma (**Figures 3A, B, D**) despite the predicted presence of IgG responses against the tested epitopes (**Figure 2C** and **Table 4**). In some instances, mutations in vulnerable epitopes resulted in a significant increase (>3 fold) in neutralization titer compared to the parent wild-type (represented in italics); the CD4bs G458Y mutation increased sensitivity of TRO.11 to neutralization by VC AA plasma (**Figure 3A**), while the MPER W672A mutation increased sensitivity of TRO.11 and COT6,15 viruses to neutralization by VC AL plasma (**Figure 3B**), and the V3-glycan ADIR.4 mutation increased sensitivity of JR-FL to neutralization by VC N, VC AA, VC AL, VC AP and AQ plasma (**Figure 3C**).

Taken together, these mapping data emphasize the polyclonal nature of the nAb response in patients with bNAb activity, as multiple known vulnerable bNAb epitopes are targeted by the polyclonal nAb response in these patients (as shown by the NFP results). Importantly, both the NFP and results with mutant viruses indicated only V3-glycan targeting neutralizing antibody responses in VC BA. In one of the VCs, VC N, there seems to be evidence of immune escape by the autologous viruses in the presence of the V3-glycan directed bnAbs.

### Negative Stain Electron Microscopy Analysis of Polyclonal VC Fab-Env Complexes Reveal CD4bs and Novel gp120-gp41 Antibody Classes in VCs

To map the dominant antibody responses in VCs with polyclonal bNAb activity, we used nsEM to image polyclonal antibody Fabs isolated from the VC plasma in complex with recombinant HIV-1 Env (BG505gp140 T332N SOSIP.664) (51). This approach (58) allowed us to circumvent the limitation of only focusing on already known targeted epitopes from non-controller (chronic progressor) studies and has the potential to identify novel classes of antibodies targeting previously unidentified epitopes.

**Figure 4:**
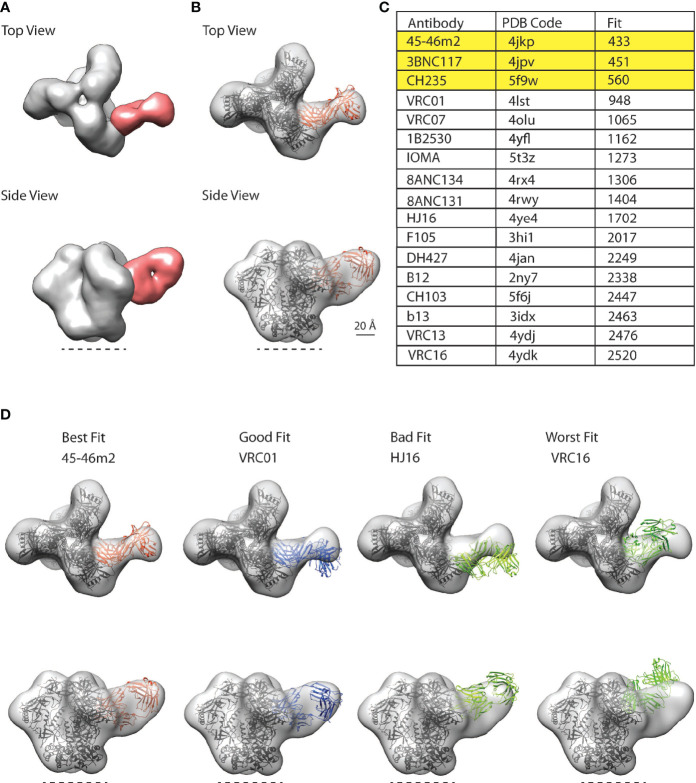
CD4bs antibody class in polyclonal plasma from VC AQ detected by nsEM. **(A)** Top and side views for representative three-dimensional (3D) reconstructions of the nsEM imaging of a mixture of VC AQ Fab (red) and BG505/T332N SOSIP.664 (grey). The dotted line indicates the viral membrane. **(B)** 3D model for the VC AQ Fab and BG505/T332N SOSIP.664 complex was rigid-body fit with a reference Fab (orange ribbon structure) bound to gp120 core aligned onto one protomer of the SOSIP trimer PDB 6myy (black ribbon structure) using UCSF Chimera’s fitmap function. The Chimera fitmap reports the number of atoms outside the EM envelope, which is indicated by ‘Fit’, with a smaller number representing a better fit. **(C)** 45-46m2 represents the best fit among the 4 CD4bs bnAbs, with the smallest Fit score. **(D)** Trimer-Fab complexes made up of a Fab (45-46m2, VRC01, HJ16 and VRC16) bound to gp120 core aligned onto one protomer of the SOSIP trimer PDB 6myy are fit into the VC AQ Fab-SOSIP nsEM (EM) map as rigid body using UCSF Chimera’s fitmap function. The Fit scores for the respective CD4bs antibodies were: 45-46m2 = 433 (best fit), VRC01 = 948 (good fit), HJ16 = 1702 (bad fit) and VRC16 = 2520 (worst fit).

A stack of 25,720 particle images consisting of bare (unliganded) and VC AQ Fab-bound SOSIPs (**Supplementary Figure 4**) was selected for 3D classification and 3D refinement, which showed the presence of a predominant CD4bs antibody class in VC AQ (**Figure 4A**). To determine whether the CD4bs antibody class in VC AQ was similar to other known CD4bs antibody classes, we fit a representative selection of available structures from the Protein Data Bank (PDB) of CD4bs antibodies bound to Env gp120 into the nsEM map from VC AQ (**Figure 4B**). The CD4bs bNAbs 45-46m2, 3BNC117 and CH235 were the most identical to the CD4bs Ab class in VC AQ, as demonstrated by their Fit scores (**Figures 4C** **, D**).

**Figure 5:**
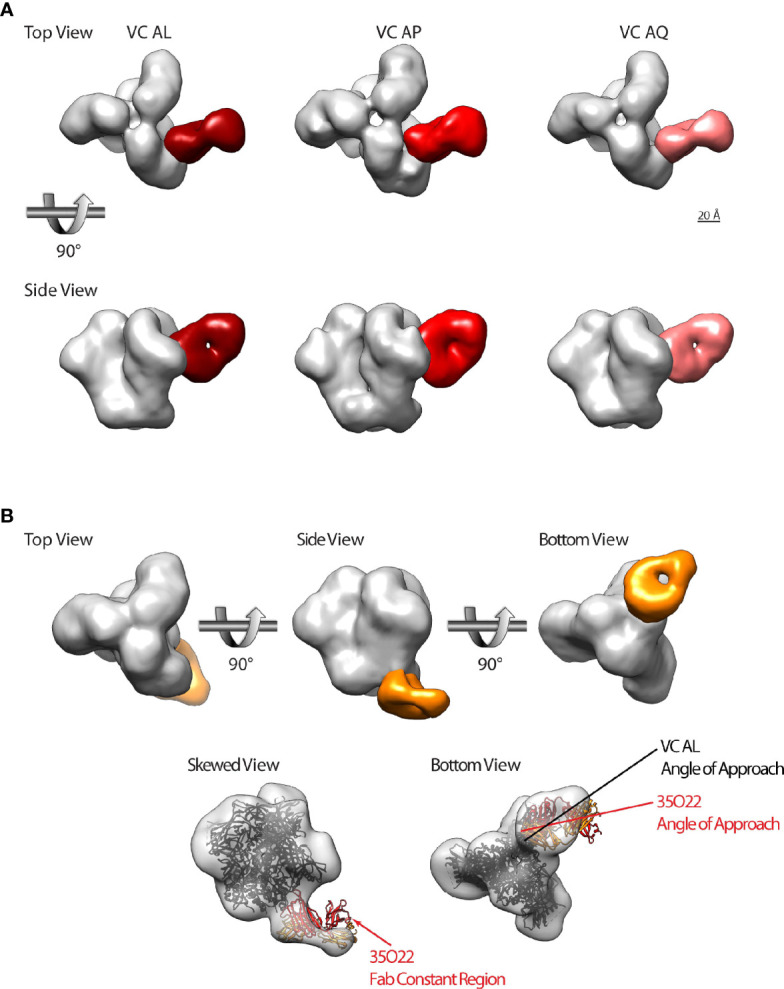
A subset of HIV-1 VCs with bNAb activity possess CD4bs and gp120-gp41 interface directed antibodies. **(A)** Top and side views of HIV-1 BG505gp140 T332N SOSIP.664 (grey) bound by Fabs that target theCD4bs from VC AL (left, dark red), VC AP (middle, red), and VC AL (right, pink). **(B)** Top row: top, side, and bottom views of HIV-1 BG505gp140 T332N SOSIP.664 (grey) bound by a VC AL Fab (orange) that targets the gp120-gp41 interface region. Bottom row: model of 35O22 Fab-bound SOSIP (ribbon diagram, PDB 5CEZ) rigidly fit into the VC AL Fab-bound EM map (transparent surface). The skewed view shows the protruding 35O22 Fab constant region, and bottom view shows the slightly different angle of approach of the VC AL and 35O22 Fabs. Similar modeling of PGT151 and 8ANC195 structures fit poorly to the VC AL Fab-bound EM map (not shown).

With the proof-of-concept showing that the nsEM epitope mapping approach can detect the dominant antibody classes in VC polyclonal plasma samples, we then interrogated VCs AL and AP together with VC AQ (**Supplementary Figure 5**) since these were the only VCs that had both bNAb activity (**Figure 1C**) and polyclonal IgG capable of mediating ADCP of BG505gp140 T332N SOSIP.664 (**Figure 2B**). Remarkably, we detected the same CD4bs antibody class (similar to the potent 45-46m2, 3BNC117 and CH235 CD4bs bnAbs) in all of the three VC patients (**Figure 5A**).

We also identified a second antibody class in VC AL that bound to the gp120-gp41 interface region vulnerable site (**Figure 5B**). Of the three gp120-gp41 interface binding antibodies with structures available (PGT151, 8ANC195 and 35O22), the VC AL Fab-bound BG505gp140 T332N SOSIP.664 was most similar to 35O22. Superimposing the structure of 35O22-bound Env onto the VC AL Fab-bound BG505gp140 T332N SOSIP.664 revealed that the epitope of the VC AL Fab-class on Env overlapped with that of 35O22, although the angle of approach of the VC AL Fab was different from that of 35O22 (**Figure 5B**).

These findings confirmed the targeting of the vulnerable CD4bs on the HIV-1 Env by the polyclonal antibody response from VCs with bNAb activity and the presence of a novel potential nAb targeting the gp120-gp41 interface.

## Discussion

We show that polyclonal bNAbs can develop in HIV-1 patients with low viral loads (<5000 copies/ml) and that these bNAbs can also mediate ADCP of primary circulating tier 2 virus strains. This finding suggests that a high viral load is not required for the development of bNAb activity. Antigenic persistence and an inflammatory environment due to low level viral replication are associated with the development of bNAbs in a low viral load setting (44). The antigenic persistence and inflammation could be involved in promoting higher levels of immune activation of monocytes, myeloid dendritic cells, and the well preserved CD4+ T and B cell compartments linked with bNAb development in VCs (59). Longitudinal studies that evaluate the ontogeny of bnAb development in VC/ECs vs. chronic viremics are needed. One hypothesis is that initial control of viremia (e.g. by CD8+ T cells) provides a low viremic environment such that when bnAbs develop they can act as part of a multi-faceted polyfunctional antiviral response to keep virus suppressed. Sajadi et al. (4) also reports on the relationship of virus levels and bnAb activity, noting that a persistent level of virus replication with plasma HIV RNA copies between 100- 10,000/ml was optimal for bnAb development.

With the aid of the novel computational modeling of the polyclonal plasma neutralization fingerprint (NFP), we show the predominant targeting of V3-glycan, CD4bs, MPER and gp120-gp41 interface bNAb epitopes by the plasma of VCs with bNAb activity. Previous studies analyzing bNAb responses in VCs have shown predominant targeting of the V3-glycan epitope (14, 15). Freund et al. showed that VC V3-glycan targeting bNAbs were potent and capable of controlling autologous viral replication in mice when co-administered with less potent CD4bs and V1V2 targeting bNAbs from the same patient (14). Analysis of contemporaneous *env* sequences from one VC patient with bNAb activity highlighted the presence of viral features suggestive of virus escape from V3-glycan-targeting bnAbs (elongated *env* V1 region and N332 to 334 glycan shift) (52–55). Thus, our findings further emphasize the targeting of the V3-glycan bNAb epitope in VCs and highlight the polyclonal nature of the bNAb response in VCs.

The polyclonality of the VC bNAb response was further emphasized by neutralization-based epitope mapping, which showed that elimination of single bNAb epitopes on viruses do not abrogate neutralization sensitivity. The polyclonal nature of the bNAb activity in these VCs (except for VC BA mono-specific targeting of the V3-glycan) potentially results in a synergistic effect that gives rise to the broad nAb response. This was also evident in a VC patient and an EC cohort from the Freund et al. (14) and Scheid et al. (60) studies, respectively. Future work isolating mAbs of different specificities from these patients will help determine their neutralization potency and their potential involvement in mediating virus neutralization either alone or in combination with other mAb specificities.

It is also plausible that targeting of novel, previously uncharacterized vulnerable epitopes on the HIV-1 Env (indicative of a unique humoral response only present in VCs) might contribute to the broad nAb response present in these patients. This theory could be indicative of a different class of antibodies present in VCs targeting a unique set of epitopes that could be more potent than the responses observed by bnAbs isolated from chronic viremics. Thus, mapping epitopes targeted by the polyclonal antibody response (both neutralizing and non-neutralizing) will provide a more complete picture of the antibody response to HIV-1.

The presence of the CD4bs bNAb antibody class in all three tested VCs with bNAbs raises the hypothesis that this antibody class, together with the V3-glycan antibody class, might be commonly elicited in VCs. CD4bs antibodies have been reported to be commonly elicited in HIV-1 infection, with VRC01-like CD4bs antibodies having been detected in 79% of a chronic viremic patient cohort from Amsterdam, 30% of a subset of CHAVI chronic viremic patients and 31% of a subset of a CAPRISA chronic viremic cohort (23). The common occurrence of this antibody class might mean that this could be an easy antibody class to target given its prevalence in not only chronic viremics but also a subset of the VCs in this study.

The similarity of the CD4bs antibody class in all three VCs with bNAbs suggests the possibility that the viruses in these patients might have the same antibody-imprinting capacity, which results in a similar predominant antibody response. The idea of virus-mediated bNAb-imprinting was described by Kuoyos et al. as being one of the determinants of bNAb evolution that could be leveraged for the development of bNAb-based HIV vaccines using immunogens based on viruses with strong bNAb-imprinting capacities (61). Analysis of the transmitter/founder viruses in the VCs with similar antibody responses will be required to confirm whether these patients have viruses that could be driving the observed similar, predominantly CD4bs antibody response.

The observed similarity of the detected VC CD4bs Abs to highly potent CD4bs bNAbs 45-46m2 [neutralizes 96% of circulating viruses (62)], CH235 [neutralizes 90% of circulating viruses (63)] and 3BNC117 [suppresses viremia after passive infusion (64)] highlighted the presence of a potent class of CD4bs Abs in these patients. NFP and BAMA epitope mapping approaches showed the presence of potent VRC01-like CD4bs bNAb in VCs AP and AQ, which was also partially supported by the structure-based nsEM epitope mapping approach. Future isolation and characterization of the ontogeny and lineages of mAbs from these CD4bs Ab classes will help shed more light on their identities and biologic activity, including the ability to neutralize contemporaneous autologous viruses. These studies will also provide more information on the previously unidentified gp120-gp41 interface antibody in VC AL, as it appears to be distinct from the bNAbs 35O22, PGT151 and 8ANC195 that target the HIV-1 Env gp120-gp41 interface. Functional characterization of the CD4bs and gp120-gp41 interface antibodies, including the role of these specificities in antibody Fc effector functions such as ADCP, will also help highlight the biologic activity of these antibody classes in VCs and whether they might be involved in virus control.

Investigating different classes of antibodies present in VC polyclonal plasma using nsEM imaging of polyclonal Fabs in complex with trimeric HIV-1 Env has some limitations, which could partially explain why we were only able to identify 2 dominant antibody classes (CD4bs and gp120-gp41 interface antibodies) in our initial analysis. The nsEM approach is known to have difficulties in picking out less frequent antibody classes (58) and also limitations in detecting V3 supersite or apex-specific antibodies when using fully glycosylated trimers, which is thought to be a result of low affinity and fast off rates of antibodies targeting these regions (58). Optimizing the nsEM approach to image more SOSIP-Fab complex particles might help increase the ns-EM imaging resolution that will result in the detection of less frequent or hard to detect antibody classes.

In summary, we discovered polyclonal bNAb activity in a subset of clade B-infected VCs. This activity was characterized by the targeting of CD4bs, V3-glycan, gp120-gp41 interface and MPER epitopes on HIV-1 Env. The VCs with bNAb activity contain a potent class of CD4bs bnAbs and a gp120-gp41 interface antibody that have not previously been reported for VCs without evidence of autoimmunity. These findings provide rationale for further testing the role of these bnAb specificities in mediating virus control in natural infection.

## Data Availability Statement

The original contributions presented in the study are included in the article/**Supplementary Material**. Further inquiries can be directed to the corresponding author.

## Ethics Statement

The studies involving human participants were reviewed and approved by Duke University IRB. The patients/participants provided their written informed consent to participate in this study.

## Author Contributions

TN and GT conceived and designed the study. TN, KM, CL, AE, and IG planned and/or performed experiments. TN and GT wrote the manuscript. RE, CL, AE, KM, DG, KJ, SD, KS, RS, LZ, SM, TH, BH, TB, IG, DM, and PA edited the manuscript. TN, RE, CL, AE, RS, LZ, and IG analyzed data. CL, SM, IG, DM, and PA contributed to experimental design and data interpretation. All authors contributed to the article and approved the submitted version.

## Funding

This work was supported by the NIH/NIAID-funded 1P01A1120756, R01AI145687, R01AI131722, R56AI052779, Center for HIV-1/AIDS Vaccine Immunology and Immunogen Discovery (Grant UMI-AI100645), and the Center for AIDS Research (P30 AI064518). 

## Conflict of Interest

The authors declare that the research was conducted in the absence of any commercial or financial relationships that could be construed as a potential conflict of interest.

## Publisher’s Note

All claims expressed in this article are solely those of the authors and do not necessarily represent those of their affiliated organizations, or those of the publisher, the editors and the reviewers. Any product that may be evaluated in this article, or claim that may be made by its manufacturer, is not guaranteed or endorsed by the publisher.

## Supplementary Material

Supplementary Figure 1 HIV-1 specific antibodies from VCs mediate ADCP and virion capture **(A)** The ability of purified IgG from HIV-1 VCs to internalize fluorescently labeled infectious tier 2 viruses (1086C, CM235 and BaL) was measured in a THP-1 based ADCP assay. Influenza-specific mAb CH65 was used as the negative control in all assays, while CH31 mAb was the positive control for the BaL and CM235 viruses, and HIVIG was a positive control for the 1086C virus. All assays were run in duplicate (%CV < 35%), and the ADCP score was calculated as described in the Materials and Methods section. Epitope exposure on the fluorescently labelled viruses BaL **(B)**, CM235 **(C)** and 1086C **(D)** was represented as a % of virus particles captured by the representative mAb (bnAbs PGT125 and VRC01, non-neutralizing 19b, 7b2 and F105 mAbs) in an infectious virus capture assay. CH65 mAb and media only (RPMI) were used as negative controls. The positivity cut-off for virus capture was calculated as 3 times the media only % capture: BaL assay % capture cut-off = 3.6%, CM235 assay capture cut-off = 4.2%, and 1086C assay capture cut-off = 23.4%. The ability of each mAb to capture virus was measured in triplicate.

Supplementary Figure 2 VCs group into high ADCP/high neutralization breadth and high ADCP/low neutralization breadth clusters. **(A)** Spearman rho was calculated for the correlation between neutralization breadth (the number of viruses neutralized with an ID_50_ greater than 30) and an ADCP breadth score (calculated by averaging the ADCP scores for BaL (Clade B), CM235 (CRF01_AE) and 1086C (Clade C) for each HIV-1 VC patient. **(B)** A hierarchical clustering algorithm (pvclust) was used to group HIV-1 VC patients based on their virion ADCP and nAb breadth scores, with the algorithm separating out the HIV-1 VCs into two significantly distinct clusters: high/high responders (high virion ADCP and high nAb breadth scores) and high/low responders (high virion ADCP and low nAb breadth scores). The Ward’s minimum variance clustering method and Euclidean distance measures were used in the hierarchical clustering approach, with a p value of <0.05 denoting statistically distinct clusters. Approximately unbiased (AU) p-values are shown in red and Bootstrap probability (BP) p-values are shown in green, with both the AU and BP p-values being 100 at the level I define the splits.

Supplementary Figure 3 Immobilized BG505/T332N SOSIP.664 exposes broadly neutralizing epitopes. Epitope exposure on the BG505/T332N SOSIP.664 was measured by testing the binding of a panel of mAbs (with and without bNAb activity) (20ug/ml) to the BG505 SOSIP.664 immobilized to streptavidin biosensors in a BLI assay. The influenza mAb CH65 was used as the negative control, and BSA immobilized to the streptavidin biosensor was used for reference (background) subtractions. This assay was done in duplicate.

Supplementary Figure 4 CD4bs antibody classes in HIV-1 VC AQ. Two-dimensional averages from the nsEM imaging of a mixture of VC AQ Fab and the BG505/T332N SOSIP.664 in 3:1 ratio resulted in the classification of 3 classes: bare SOSIP (L), and two Fab-bound SOSIP classes (R). The two Fab-bound classes are essentially identical, within limits of resolution (~20 A) and hence the two Fab-bound classes were combined and used for final 3D refinement.

Supplementary Figure 5 SEC purified VC AL, VC AP and VC AQ Fab-bound HIV-1 BG505gp140 T332N SOSIP.664 **(A)** HIV-1 VC Fab complexed with BG505gp140 T332N SOSIP.664 was purified from unbound HIV-1 VC Fab and BG505gp140 T32N SOSIP.664 fractions using size exclusion chromatography (SEC). The first peak (peak 1) represented the fraction containing the HIV-1 VC Fab complexed with BG505gp140 T332N SOSIP.664, whereas the second (peak 2) and third (peak 3) peaks represent the unbound BG505gp140 T332N SOSIP.664 and HIV-1 VC Fab, respectively. The VC AQ SEC profile has an extra peak (peak 4) due to the presence of Fc fragments. **(B)** Unreduced SDS-PAGE showing the fractions of the SEC eluted peaks (peaks 1 -4) from VCs AL, AP and AQ. BG505gp140 T332N SOSIP.664 (BG505 SOSIP) and purified Fab fraction proteins were used as controls. The thick band in the Peak 1 fraction represents the HIV-1 VC Fab complexed with BG505gp140 T332N SOSIP.664, while the light band represents just the HIV-1 VC Fab.

Supplementary Table 1 HIV-1 Env Pseudotyped Global Virus Panel. Pseudotyped HIV-1 viruses representative of the global circulating HIV-1 strains used in the TZM-bl neutralization assay (40). Clade B MN.3 was used as a tier 1A reference strain.

Supplementary Table 2 Neutralization Fingerprinting (NFP) Virus Panel. A 30-strain HIV-1 virus panel optimized for sequence diversity and the ability to correctly cluster the neutralization fingerprints of reference bnAbs according to epitope specificity and high minimum bnAb neutralization breadth (32).
