# Supplementary figures and images for: Polyclonal Broadly Neutralizing Antibody Activity Characterized by CD4 Binding Site and V3-Glycan Antibodies in a Subset of HIV-1 Virus Controllers
Source: Front Immunol. 2021 Dec 23;12:670561. doi: 10.3389/fimmu.2021.670561 (PMC8733328; doi:10.3389/fimmu.2021.670561)

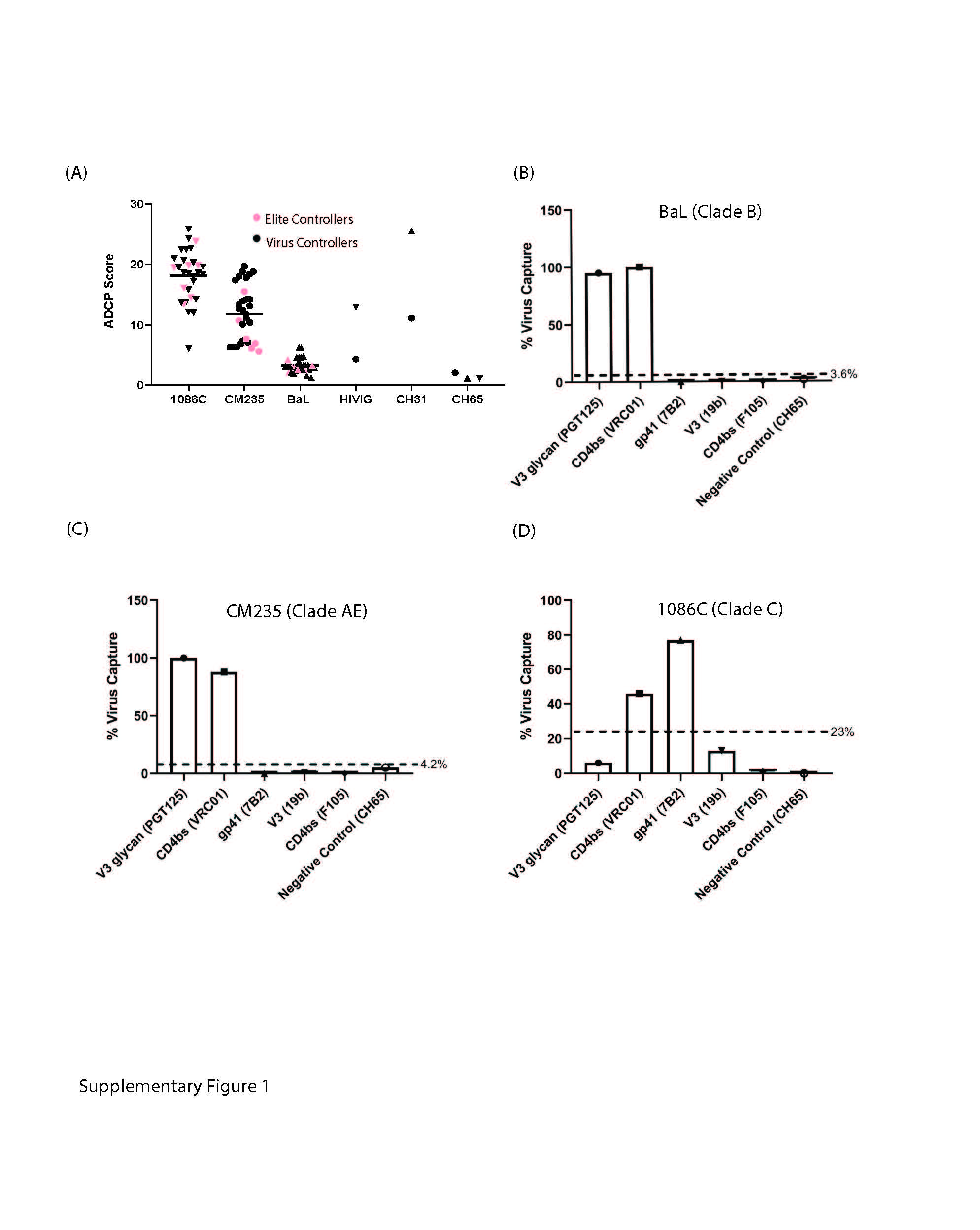

Supplement: Supplementary Figure 1 — HIV-1 specific antibodies from VCs mediate ADCP and virion capture (A) The ability of purified IgG from HIV-1 VCs to internalize fluorescently labeled infectious tier 2 viruses (1086C, CM235 and BaL) was measured in a THP-1 based ADCP assay. Influenza-specific mAb CH65 was used as the negative control in all assays, while CH31 mAb was the positive control for the BaL and CM235 viruses, and HIVIG was a positive control for the 1086C virus. All assays were run in duplicate (%CV < 35%), and the ADCP score was calculated as described in the Materials and Methods section. Epitope exposure on the fluorescently labelled viruses BaL (B), CM235 (C) and 1086C (D) was represented as a % of virus particles captured by the representative mAb (bnAbs PGT125 and VRC01, non-neutralizing 19b, 7b2 and F105 mAbs) in an infectious virus capture assay. CH65 mAb and media only (RPMI) were used as negative controls. The positivity cut-off for virus capture was calculated as 3 times the media only % capture: BaL assay % capture cut-off = 3.6%, CM235 assay capture cut-off = 4.2%, and 1086C assay capture cut-off = 23.4%. The ability of each mAb to capture virus was measured in triplicate. [file Image_1.jpeg]

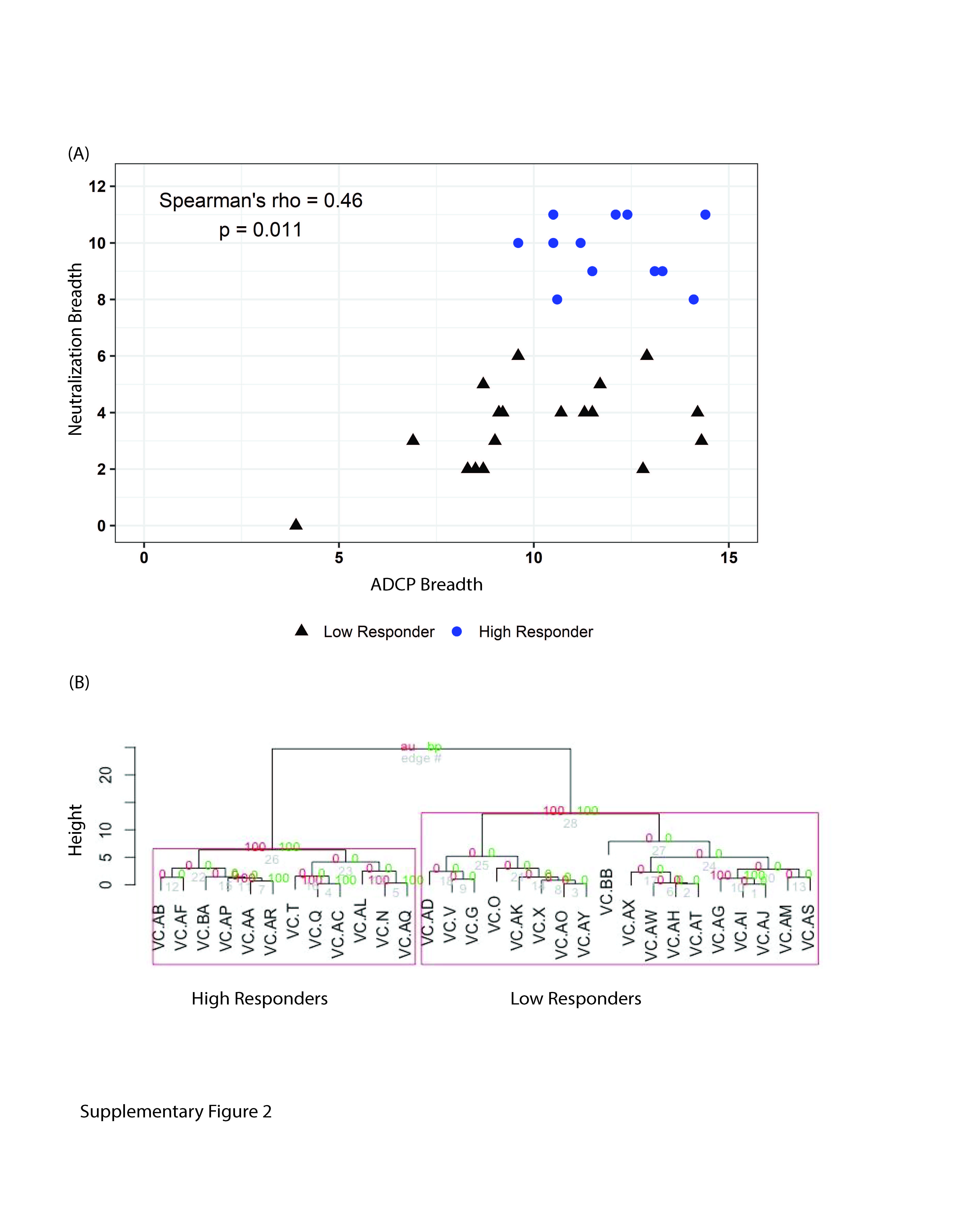

Supplement: Supplementary Figure 2 — VCs group into high ADCP/high neutralization breadth and high ADCP/low neutralization breadth clusters. (A) Spearman rho was calculated for the correlation between neutralization breadth (the number of viruses neutralized with an ID50 greater than 30) and an ADCP breadth score (calculated by averaging the ADCP scores for BaL (Clade B), CM235 (CRF01_AE) and 1086C (Clade C) for each HIV-1 VC patient. (B) A hierarchical clustering algorithm (pvclust) was used to group HIV-1 VC patients based on their virion ADCP and nAb breadth scores, with the algorithm separating out the HIV-1 VCs into two significantly distinct clusters: high/high responders (high virion ADCP and high nAb breadth scores) and high/low responders (high virion ADCP and low nAb breadth scores). The Ward’s minimum variance clustering method and Euclidean distance measures were used in the hierarchical clustering approach, with a p value of <0.05 denoting statistically distinct clusters. Approximately unbiased (AU) p-values are shown in red and Bootstrap probability (BP) p-values are shown in green, with both the AU and BP p-values being 100 at the level I define the splits. [file Image_2.jpeg]

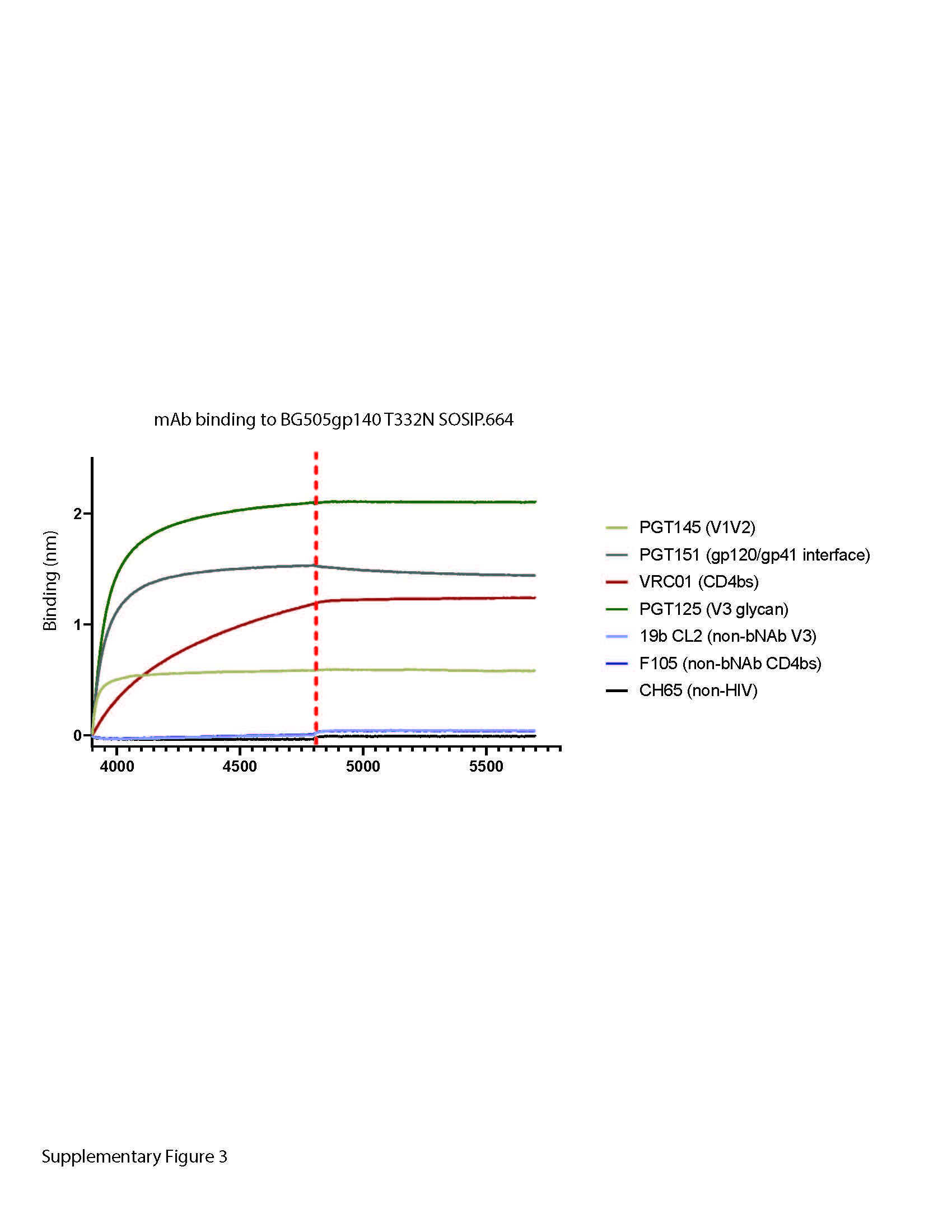

Supplement: Supplementary Figure 3 — Immobilized BG505/T332N SOSIP.664 exposes broadly neutralizing epitopes. Epitope exposure on the BG505/T332N SOSIP.664 was measured by testing the binding of a panel of mAbs (with and without bNAb activity) (20ug/ml) to the BG505 SOSIP.664 immobilized to streptavidin biosensors in a BLI assay. The influenza mAb CH65 was used as the negative control, and BSA immobilized to the streptavidin biosensor was used for reference (background) subtractions. This assay was done in duplicate. [file Image_3.jpeg]

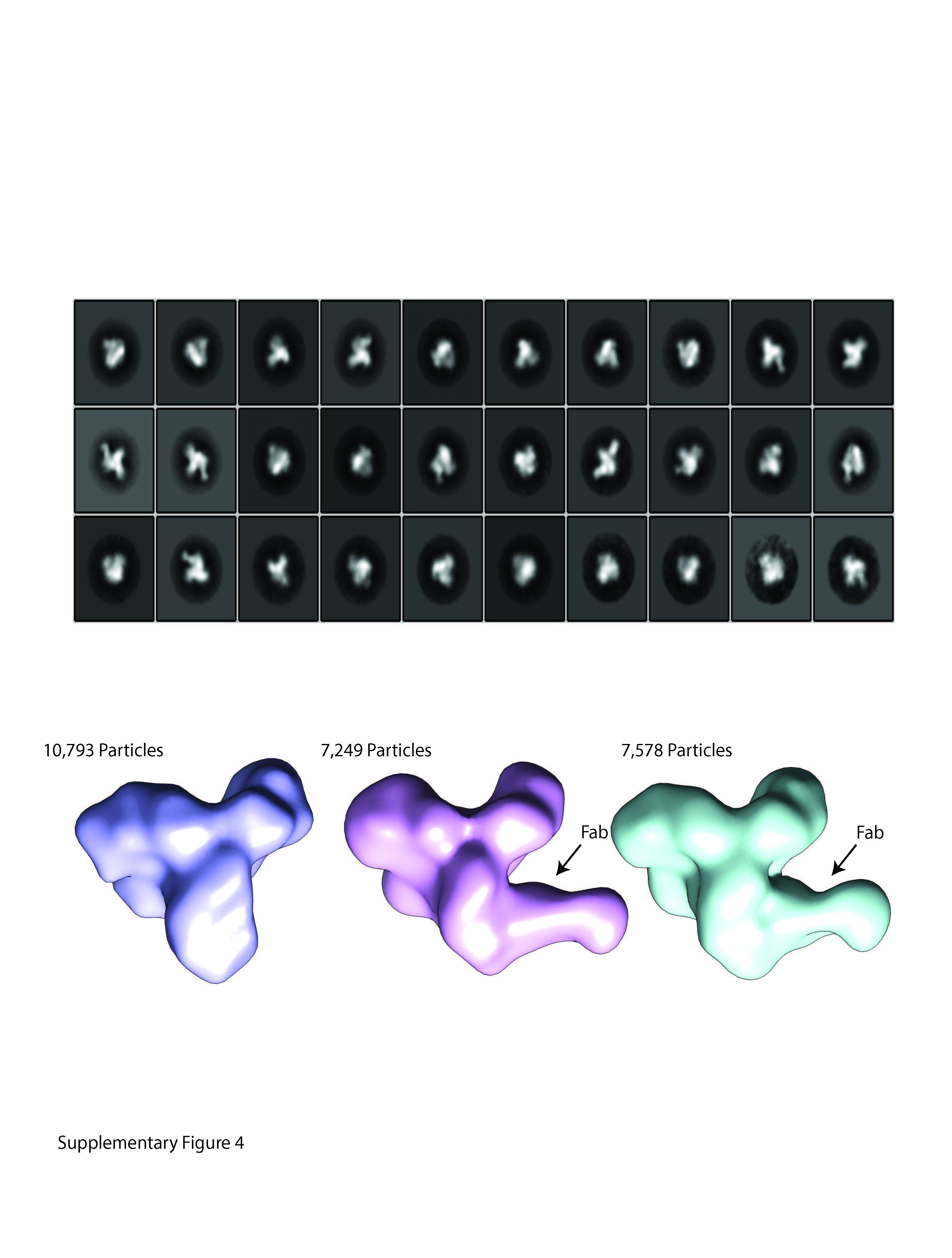

Supplement: Supplementary Figure 4 — CD4bs antibody classes in HIV-1 VC AQ. Two-dimensional averages from the nsEM imaging of a mixture of VC AQ Fab and the BG505/T332N SOSIP.664 in 3:1 ratio resulted in the classification of 3 classes: bare SOSIP (L), and two Fab-bound SOSIP classes (R). The two Fab-bound classes are essentially identical, within limits of resolution (~20 A) and hence the two Fab-bound classes were combined and used for final 3D refinement. [file Image_4.jpeg]

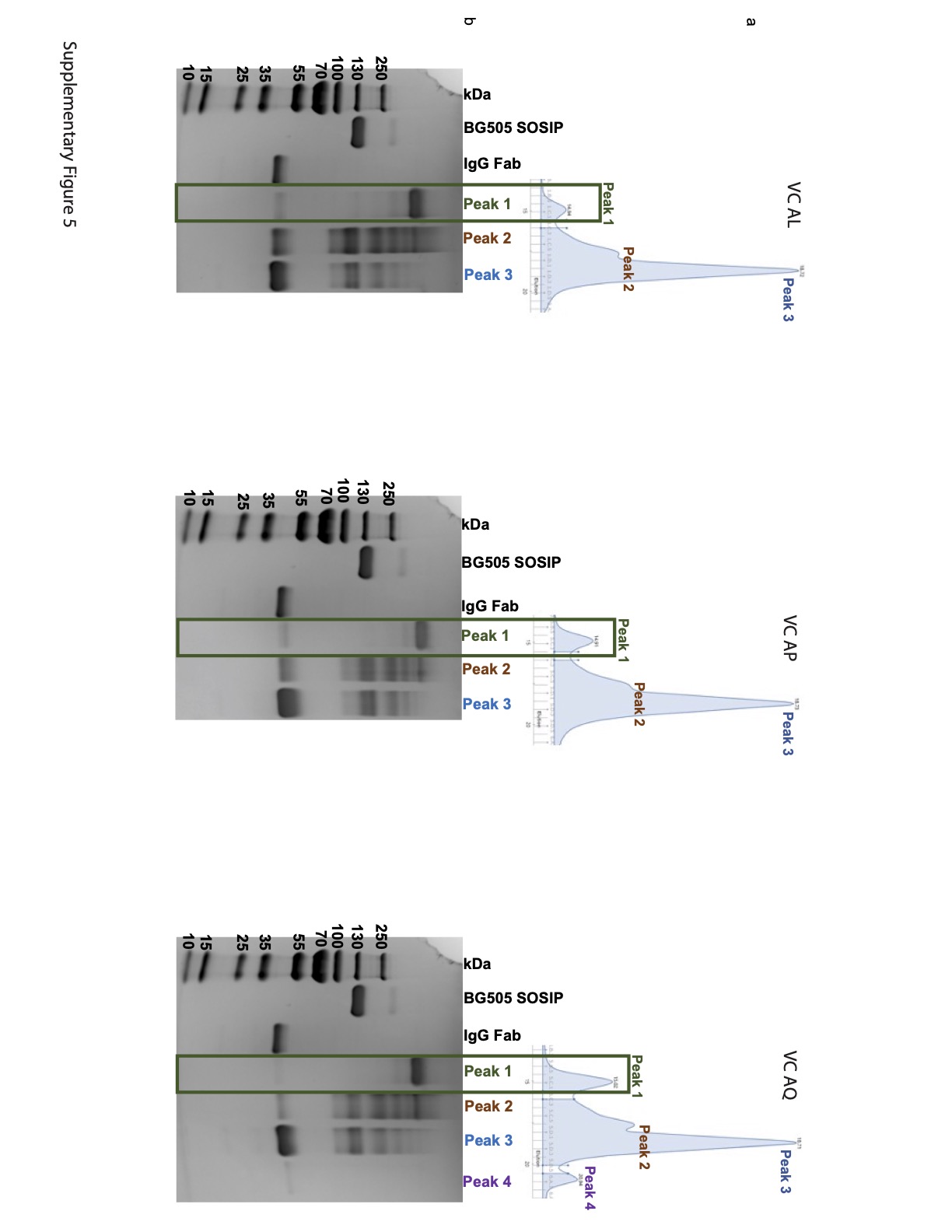

Supplement: Supplementary Figure 5 — SEC purified VC AL, VC AP and VC AQ Fab-bound HIV-1 BG505gp140 T332N SOSIP.664 (A) HIV-1 VC Fab complexed with BG505gp140 T332N SOSIP.664 was purified from unbound HIV-1 VC Fab and BG505gp140 T32N SOSIP.664 fractions using size exclusion chromatography (SEC). The first peak (peak 1) represented the fraction containing the HIV-1 VC Fab complexed with BG505gp140 T332N SOSIP.664, whereas the second (peak 2) and third (peak 3) peaks represent the unbound BG505gp140 T332N SOSIP.664 and HIV-1 VC Fab, respectively. The VC AQ SEC profile has an extra peak (peak 4) due to the presence of Fc fragments. (B) Unreduced SDS-PAGE showing the fractions of the SEC eluted peaks (peaks 1 -4) from VCs AL, AP and AQ. BG505gp140 T332N SOSIP.664 (BG505 SOSIP) and purified Fab fraction proteins were used as controls. The thick band in the Peak 1 fraction represents the HIV-1 VC Fab complexed with BG505gp140 T332N SOSIP.664, while the light band represents just the HIV-1 VC Fab. [file Image_5.jpeg]
