# Supplementary material for: Polyclonal Broadly Neutralizing Antibody Activity Characterized by CD4 Binding Site and V3-Glycan Antibodies in a Subset of HIV-1 Virus Controllers
Source: Front Immunol. 2021 Dec 23;12:670561. doi: 10.3389/fimmu.2021.670561 (PMC8733328; doi:10.3389/fimmu.2021.670561)
Supplement: Supplementary Table 1 — HIV-1 Env Pseudotyped Global Virus Panel. Pseudotyped HIV-1 viruses representative of the global circulating HIV-1 strains used in the TZM-bl neutralization assay (40). Clade B MN.3 was used as a tier 1A reference strain. [file Table_1.docx]

**Supplementary Tables_Nyanhete et al.**

**Supplemental Table 1: HIV-1 Env Pseudotyped Global Virus Panel.** Pseudotyped HIV-1 viruses representative of the global circulating HIV-1 strains used in the TZM-bl neutralization assay **(deCamp et al., 2014)**. Clade B MN.3 was used as a tier 1A reference strain.

| **HIV Envelope** | **Subtype** | **Tier** | **Country of Origin** |
| --- | --- | --- | --- |
| TRO11 | B | 2 | Italy |
| 25710 | C | 2 | India |
| X2278 | B | 1B | Spain |
| BJOX2000 | CRF07_BC | 2 | China |
| X1632 | G | 2 | Spain |
| CE1176 | C | 2 | Malawi |
| 246F3 | AC | 2 | Tanzania |
| CH119 | CRF07_BC | 2 | China |
| CE0217 | C | 2 | Malawi |
| CNE55 | CRF01_AE | 2 | China |

**Supplemental Table 2: Neutralization Fingerprinting (NFP) Virus Panel**. A 30-strain HIV-1 virus panel optimized for sequence diversity and the ability to correctly cluster the neutralization fingerprints of reference bnAbs according to epitope specificity and high minimum bnAb neutralization breadth **(Doria-Rose et al., 2017)**.

| **Virus Name** | **Clade** |
| --- | --- |
| 7165.18 | B |
| 16845-2.22 | C |
| 231965.c01 | D |
| T235-47 | AG |
| 26191-2.48 | C |
| T235-8 | AG |
| 3168_V4_C10 | C |
| 620345.c01 | AE |
| 6785_V5_C14 | C |
| C1080.c03 | AE |
| CH038.12 | BC |
| CH181.12 | BC |
| CNE5 | AE |
| CNE55 | AE |
| DU123.6 | C |
| DU422.1 | C |
| Q259.d2.17 | A |
| Q461.e2 | AD |
| QH0515.1 | B |
| QH0692.42 | B |
| R1166.c01 | AE |
| R2184.c04 | AE |
| RHPA4259.7 | B |
| T257-31 | AG |
| TRO.11 | B |
| WITO4160.33 | B |
| X2088_c9 | G |
| ZM135M.PL10a | C |
| ZM249M.PL1 | C |
| ZM53M.PB12 | C |
